# Phytochemical Analysis and Study of Antioxidant, Anticandidal, and Antibacterial Activities of *Teucrium polium* subsp. *polium* and *Micromeria graeca* (Lamiaceae) Essential Oils from Northern Morocco

**DOI:** 10.1155/2021/6641720

**Published:** 2021-03-13

**Authors:** Taoufiq Benali, Khaoula Habbadi, Abdelhakim Bouyahya, Abdelmajid Khabbach, Ilias Marmouzi, Tarik Aanniz, Houda Chtibi, Hanae Naceiri Mrabti, El Hassan Achbani, Khalil Hammani

**Affiliations:** ^1^Environment and Health Team, Polydisciplinary Faculty of Safi, Cadi Ayyad University, Marrakesh, Morocco; ^2^Laboratory of Natural Resources and Environment, Polydisciplinary Faculty of Taza, Sidi Mohamed Ben Abdellah University, B.P.: 1223, Taza-Gare, Taza, Morocco; ^3^Laboratoire de Recherche et de Protection des Plantes URPP- INRA-Meknès, Meknès, Morocco; ^4^Laboratory of Human Pathologies Biology, Department of Biology, Faculty of Sciences, and Genomic Center of Human Pathologies, Faculty of Medicine and Pharmacy, Mohammed V University in Rabat, Rabat, Morocco; ^5^Laboratory of Materials, Natural Substances, Environment and Modeling, Polydisciplinary Faculty of Taza, Sidi Mohamed Ben Abdellah University, B.P.: 1223, Taza-Gare, Taza, Morocco; ^6^University Mohammed V in Rabat, Faculty of Medicine and Pharmacy, Laboratory of de Pharmacology et Toxicology, Rabat Instituts, BP 6203, Rabat, Morocco; ^7^Medical Biotechnology Laboratory (MedBiotech), Rabat Medical & Pharmacy School, Mohammed V University in Rabat, Rabat 6203, Morocco; ^8^Laboratory of Pharmacology and Toxicology, Bio Pharmaceutical and Toxicological Analyzes Research Team, Faculty of Medicine and Pharmacy, Mohammed V University in Rabat, BP 6203, Rabat, Morocco

## Abstract

The protection of agricultural crops and the preservation of the organoleptic and health qualities of food products represent a major challenge for the agricultural and agro-food industries. Essential oils have received greater attention as alternatives to replace the control strategies based on pesticides against phytopathogenic bacteria and synthetic compounds in food preservation. The aims of this work were to study the chemical composition of *Teucrium polium* subsp. *polium* and *Micromeria graeca* essential oils and to examine their antioxidant and antimicrobial effects. To carry out this work, the chemical composition of the essential oil was determined using gas chromatography (GC) with the detection feature of mass spectrometry (MS). Subsequently, the antioxidant activity was investigated by DPPH and FRAPS assays. The antimicrobial effect was studied against phytopathogenic and foodborne pathogenic bacteria using the disc and the microdilution methods. Our results showed that GC-MS analysis of EOs allowed the identification of 30 compounds in *T. polium* EO (TPpEO), while 5 compounds were identified in *M. graeca* EO (MGEO). TPpEO had as major compounds *β*-pinene (19.82%) and germacrene D (18.33%), while geranial (36.93%) and z-citral (18.25%) were the main components of MGEO. The most potent activity was obtained from MGEO (IC_50_ = 189.7 ± 2.62 *µ*g/mL) compared to TPpEO (IC_50_ = 208.33 ± 3.51 *µ*g/mL. For the FRAP test, the highest reducing power was obtained from 1.32 ± 0.1 mg AAE/g of TPpEO compared to MGEO 0.51 ± 0.13 mg AAE/g of EO. Both EOs exhibited varying degrees of antibacterial activities against all the tested strains with inhibition zones in the range of 9.33 ± 0.57 mm to >65 mm and MIC values from 0.19 to 12.5 mg/mL. However, MGEO exhibits an interesting anticandidal effect with inhibition zone 44.33 ± 0.57 mm. The findings of this research establish the riches of EOs on volatile compounds, their important antioxidant activity, and their antimicrobial effect against the bacteria tested.

## 1. Introduction

The security of agricultural crops and both organoleptic and health qualities of food products represent a main defiance for the agricultural and agro-food industries [1, 2]. The control of the problems caused by phytopathogenic bacteria is based on use of pesticides and antibiotics with potential side effects on the environment and living beings. Such chemicals are not very biodegradable and represent a risk of developing antibiotic resistance, which inspired the European Union to limit their use [3, 4]. On the other hand, the preservation of food products is assured by synthetic compounds called “food additives,” presented potential side effects on the consumer [5–7]. The plant extracts and essential oils as antioxidant and antimicrobial agents are focused to overcome these problems and to satisfy the improved demand for more natural solutions.

For a long time, different cultures and civilizations worldwide have been using plants as drugs to treat numerous diseases [8–10]. The essential oils (EOs) are among the natural products of great interest in food, cosmetic, and pharmaceutical industries due to their antioxidant and antibacterial [11–18], antifungal [19–25], antiparasitic [6, 26], insecticidal [27–31], and anticancer activities [32–34].

Morocco by its biogeographical position is characterized, on the one hand, by ecological and floristic diversities and, on the other hand, by a long tradition and expertise in the use of plant medicines [35–37]. Previous works in some regions of Morocco have shown that the Moroccan pharmacopoeia is dominated mainly by *Lamiaceae* [38–41]. A great economic importance is given to many of their species due to their EO production [42] and their traditional use [40, 43, 44]. In recent decades, in the goal to valorize the Moroccan *Lamiaceae* species, previous researchers have evaluated the antioxidant and antimicrobial activities of essential oils of many plants [45–49]. In this order, our study focused on two species of *Lamiaceae*, *Teucrium polium* subsp*. polium* and *Micromeria graeca*, locally known as “Jaâda” and “Bakolt'nhal,” respectively. These species have been strongly used in Moroccan traditional medicine [38–40, 44, 50].

To the best of our knowledge, no reports on the variation of essential oil composition and biological activities of these plants collected from the Province of Taza, Northern Morocco, are available. Therefore, the objectives of this study were the identification of volatile compounds of hydrodistilled EOs of *T. polium* and *M. graeca* and the investigation of their antioxidant and antimicrobial activities.

## 2. Materials and Methods

### 2.1. Collection of Plants and Isolation of Essential Oils

Both plants were collected in April 2016 from the Province of Taza, Northern Morocco (004° 52.607′ N, 004°01.190′ W and 34°09.825′ N, 004°09.850′ W). The identification of plants was achieved by Pr. Ennabili Abdeslam and Dr Khabbach Abdelmajid in the Natural Resources and Environment Laboratory of the Polydisciplinary Faculty of Taza, Sidi Mohamed Ben Abdellah University of Fez, where a voucher plant specimen has been deposited for future reference (FPT-LRNE-73: *Teucrium polium* subsp. *polium* and FPT-LRNE-72: *Micromeria graeca*). The aerial parts of the plants were dried at room temperature. Then, the plant sample (100 g) was subjected to hydrodistillation using a Clevenger-type apparatus for 4 h. The essential oil was stored at 4°C until use.

### 2.2. Gas Chromatography-Mass Spectrometry (GC-MS) Analysis

The chemical composition of EOs was analyzed according the conditions described in our previous works [51, 52]. For each compound, the Kovats retention index (RI) was calculated relative to a standard mix of n-alkanes between C9 and C31 (Sigma-Aldrich Co.). Identification of constituents was performed by comparison of RI and MS spectra with those reported in the literature and by computer matching with standard reference databases (NIST98, Wiley275, and CNRS libraries).

### 2.3. Antioxidant Activity

#### 2.3.1. Free Radical Scavenging Activity

The radical effect of EOs was evaluated using the radical 2.2-diphenyl-1-picrylhydrazyl (DPPH) as reported by Benali et al. [52] and Huang et al. [53], with some modifications. In brief, the DPPH solution (0.2 mM in methanol) was prepared. Then, 2.5 mL of test sample at different concentrations (2.5–100 *μ*g/mL) was added to 0.5 mL of DPPH solution, and the absorbance of samples was measured at 517 nm after 30 min. Ascorbic acid and Trolox were used as positive controls.

The calculation of the antioxidant activity was done according to the following formula:

(1) $$\begin{array}{c} \text{DPPH scavenging activity }\left(\%\right)=\left[\frac{\left(A_{0}-A_{s}\right)}{A_{0}}\right]{}^{*}100, \end{array}$$

where *A*_**0**_ is the absorbance of the negative control and **A**_**s**_ is the absorbance of the test sample at 30 min. The test was carried out in triplicate, and the IC_50_ values were reported as mean ± SD.

#### 2.3.2. Reducing Power of Ferric Ions

The reducing activity of EOs was determined according to Benali et al. [52] and Oyaizu [54]. The mixture of the sample (1 mL), the phosphate buffer (2.5 mL, 0.2 M, pH 6.6), and the potassium ferricyanide (2.5 mL) was prepared. After incubation for 20 min at 50°C (water bath), 2.5 mL of trichloroacetic acid (10%) was added to the mixture. Then, the solution was centrifuged at 3000 Trs/min for 10 min. Finally, 2.5 mL of the supernatant was mixed with 2.5 mL of distilled water and 0.5 mL of 0.1% ferric chloride. Absorbance was measured at 700 nm.

Ascorbic acid (50–450 *μ*g/mL) is used as a standard. The reducing power is expressed in milligram equivalence of ascorbic acid per gram of essential oil (mg AAE/g of EO).

### 2.4. Antimicrobial Activity

#### 2.4.1. Microorganism Strains, Origin, and Growth Conditions

The foodborne pathogenic bacteria used including Gram-positive (*Listeria innocua CECT 4030*, *Staphylococcus aureus CECT 976*, and *Bacillus subtilis DSM 6633*) and Gram-negative (*Proteus mirabilis*, *Escherichia coli K12*, and *Pseudomonas aeruginosa CECT 118*) bacteria were obtained from the Laboratory of Biology and Health, Sciences Faculty of Tetouan; *Candida albicans ATCC 10231* was, also, used which was obtained from the Laboratory of Agri-Food and Health, Sciences and Technics Faculty of Settat, Morocco. Plant pathogenic bacteria were *Clavibacter michiganensis* subsp*. michiganensis 1616-3* and *Pseudomonas savastanoi* pv*. savastanoi* (PSS2636-40) which were obtained from the Laboratory of Researches and Protection of Plants, URPP- INRA-Meknes, Morocco.

The pathogen bacterial strains were cultivated in Mueller-Hinton agar (MHA) or Mueller-Hinton Broth (MHB) at 37°C for 24 h as described by Benali et al. [52]. The fungi and the phytopathogenic bacteria were cultured in YPGA medium (5 g yeast extract, 5 g peptone, 10 g glucose, 15–18 g agar, in 1 liter) or YPG and incubated as following: 48 h at 37°C for *Candida albicans* ATCC 10231; 48 h at 25°C for *Pseudomonas savastanoi* pv*. savastanoi 636-40*; 72 h at 25°C for *Clavibacter michiganensis* subsp*. michiganensis* 1616–3. The inoculum test concentrations are 10^6^ CFU/mL for bacteria, 10^8^ CFU/mL for phytopathogenic plant, and 10^5^ spores/mL for fungi.

#### 2.4.2. Antimicrobial Activity

The antibacterial activity was evaluating using disc diffusion method as described by Benali et al. [52] and Rota et al. [55], with some modifications. In brief, sterile disks (6 mm diameter) containing 12.5 *µ*L of pure essential oil were applied onto the surface of the agar medium which were previously spread by the test inoculum concentrations. Gentamicin (15 *μ*g), vancomycin (30 *µ*g), streptomycin (25 *μ*g), and amphotericin (10 *μ*g) were used as a positive control. Negative control consisted of 10% dimethylsulfoxide (DMSO). After incubation as described above, the antimicrobial activity was assessed by measuring the diameter of inhibition zones. Tests were performed in triplicate.

#### 2.4.3. Determination of Minimum Inhibitory Concentration

MIC was determined only for strains considered very sensitive and essential oils considered very active leading to diameters larger than 15 mm [52–56]. Minimum inhibitory concentrations (MICs) were realized in sterile 96-well microplate as described by Güllüce et al. [22], with some modifications. First, 100 *μ*L of MHB was distributed in all test wells, except the first well in which a volume of 200 *μ*L containing the essential oil at a concentration of 25 mg/mL in 10% DMSO. A series of concentrations ranging from 0.097 to 25 mg/mL were prepared by the transfer of 100 *μ*L by scalar dilutions from the first to the ninth well. Then, except the 10th well used as sterility control, 10 *μ*L of the suspension from each well was removed and replaced by the test inoculum concentrations as described above. The eleventh well was considered as positive growth control containing only broth medium. The last well containing 10% DMSO (v/v), without oils, was used as negative control. Then, the plates were incubated at conditions of growth as described above. After incubation, a volume of 25 *μ*L of an indicator of microorganism's growth was added in each well, and tetrazolium (MTT: 3-(4,5-dimethythiazol)-2-yl-2, 5-diphenyltetrazolium bromide (Sigma)) was prepared at a concentration of 0.5 mg/mL in sterile distilled water. The microplate was re-incubated for 30 min at temperature 25°C or 37°C. Where microbial growth was inhibited, the solution keeps the initial color of MTT. To determine the minimum bactericidal concentration (MBC) value, 10 *μ*L of broth from the uncolored wells was inoculated and incubated at growth conditions.

### 2.5. Statistical Analysis

All experiments were done in triplicates and values of each were expressed as mean ± standard deviation (SD) and were subjected to analysis of variance (one-way ANOVA). The statistical analysis was performed using GraphPad Prism version 6.00 (GraphPad Inc., San Diego, California). Differences (between groups) were considered as statistically significant at *p* < 0.05.

## 3. Results

### 3.1. Chemical Composition

**Figure 1:**
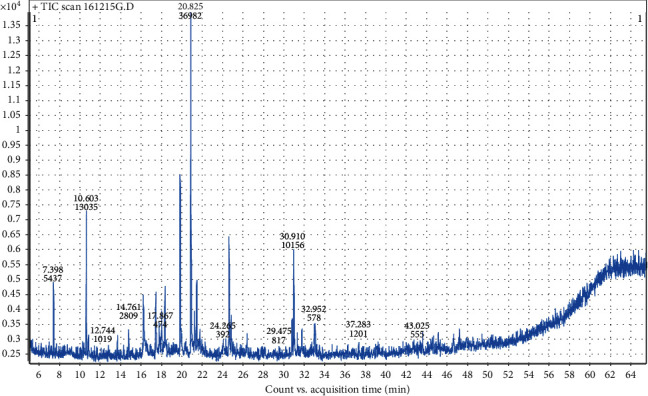
GC analysis of *Micromeria graeca* essential oil.

**Figure 2:**
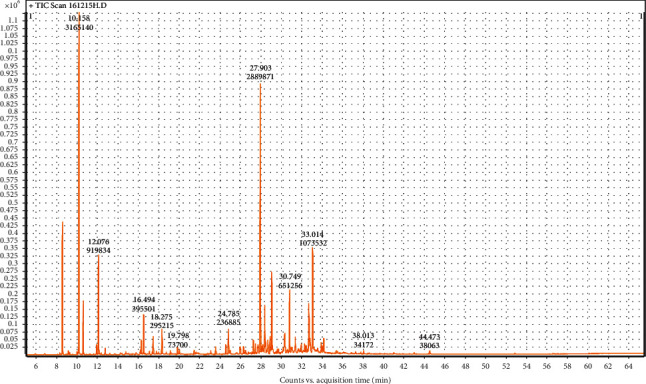
GC analysis of *Teucrium polium* subsp. *polium* essential oil.

The essential oil yields (w/w) were 0.24 ± 0.02% and 0.18 ± 0.02%, for *Micromeria graeca* and *Teucrium polium* subsp*. polium*, respectively. Volatile compounds of both studied plants were separated by GC (Figures 1 and 2) and identified using MS analysis. The results obtained by GC-MS analysis of EOs are summarized in Table 1. As summarized, 29 and 5 compounds were identified in TPpEO and MGEO representing 97.46% and 99.95% of the total, respectively. Our results showed that the major compounds in TPpEO are *β*-pinene (19.82%), germacrene D (18.33%), *α*-cadinol (6.83%), *α*-pinene (6.76%), limonene (5.71%), epi-bicyclosesquiphellandrene (5.05%), delta-cadinene (4.51%), spathulenol (4.15%), bicyclogermacrene (3.21%), myrcene (2.9%), and camphor (2.45%). However, MGEO contains geranial (36.93%) as a main component followed bay *z*-citral (18.25%), 1,8-epoxy-p-menth-2-ene (13.01%), nerol (11.96%), and isoaromadendrene epoxide (10.14%).

### 3.2. Antioxidant Activity

The essential oils were evaluated for their antioxidant effect using two methods, the DPPH free radical scavenging and the ferric ion reduction assay (FRAP). For the DPPH assay, as summarized in Table 2, the most potent activity was obtained from *M. graeca* (IC_50_ = 189.7 ± 2.62 *µ*g/mL), followed by *T. polium* (IC_50_ = 208.33 ± 3.51 *µ*g/mL), but they were all less potent than the standards used as positive controls, namely, Trolox and ascorbic acid (IC_50_ = 1.4 ± 0.04 *µ*g/mL and IC_50_ = 1.82 ± 0.025 *µ*g/mL, respectively). For the FRAP test, the results were expressed in milligram equivalence of ascorbic acid per gram of extract (mg AAE/g of EO), and the highest reducing power was obtained from TPpEO 1.32 ± 0.1 mg AAE/g of EO compared to MGEO 0.51 ± 0.13 mg AAE/g of EO.

### 3.3. Antimicrobial Activity

The *in vitro* antimicrobial activity of the essential oils against the tested microorganisms was qualitatively and quantitatively confirmed by diameter of inhibition zone and the MIC values. As shown in Tables 3 and 4, the essential oils exhibited varying degrees of antibacterial activity against all tested strains. For the essential oil of TPpEO, the inhibition zones were in the range from 7.33 to 52 mm, with MIC values of 0.19 mg/mL and 0.78 mg/mL. *C. michiganensis* was the most sensitive bacteria to TPpEO with inhibition zone of 52 ± 1 mm with MIC value of 0.78 mg/mL, followed by *B. subtilis* (23 ± 2 mm), *P. savastanoi PSS2636-40* (22 ± 1 mm), and *P. mirabilis* (21.33 ± 2.08 mm). This oil has a low effect against the other bacteria. No antifungal activity is observed against *C. albicans* (7.33 ± 0.57 mm).

For MGEO, the inhibition zones varied from 9.33 to >65 mm, with MIC values from 0.19 to 12.5 mg/mL. *C. michiganensis* was the most sensitive bacteria with inhibition zone superior to 65 mm with MIC value of 0.19 mg/mL, followed by *P. savastanoi PSS2636-40* (49 ± 1 mm), *B. subtilis* (28.33 ± 1.52 mm), *S. aureus* (22 ± 1 mm), *P. mirabilis* (20 ± 2 mm), *L. innocua* (19.33 ± 1.15 mm), and *E. coli K12* (17.66 ± 1.52 mm). For antifungal activity, MGEO exhibits a good anticandidal effect with an inhibition zone of 44.33 ± 0.57 mm and MIC value of 3.12 mg/mL compared to control positive amphotericin (18.66 ± 1.15 mm).

## 4. Discussion

TPpEO and MGEO aerial parts showed qualitative and/or quantitative variability in chemical composition when compared with other reports. The GC-MS analysis of TPpEO and MGEO aerial parts showed that the present finding is similar to those of Algerian *T. polium subsp. polium* that demonstrated germacrene (14.8%) *β*-pinene (16.6%), and *α*-pinene (7.2%) as main compounds [57], except for *α*-cadinol (6.83%), epi-bicyclosesquiphellandrene (5.05%), *δ*-cadinene (4.51%), and camphor (2.45%), which were not detected in the Algerian sample. However, the results are different to the only one investigation of oil analysis of Moroccan *T. polium* subsp. *polium* from the regions of Midelt, which indicated 3-carene (16.49%), *γ*-muurolene (14.03%), *α*-pinene (9.94%), *α*-phellandrene (6.93%), and caryophyllene (7.51%) as major constituents [58]. The results of the volatile product analysis of *Teucrium polium* species from Saudi Arabia, Algeria, Jordan, Greece, Turkey, and Serbia identified the following compounds with a high content: *β*-pinene, limonene, germacrene D, *α*-pinene, bicyclogermacrene, and spathulenol [59–64].

For MGEO from Morocco, this is the first study of their chemical composition. In Greece, EOs of two samples of this plant were characterized by the presence of caryophyllene oxide (17.0%), epi-*α*-bisabolol (12.8%), linalool (18.1%), and *β*-chamigrene (12.5%) [65]. Compared with other species of the *Micromeria* genus, the study of the chemical composition of *Micromeria cilicica* EO from Tukey showed that the major components characterized were pulegone, cis-p-menthone, and trans-p-menthone [66]. In addition, *Micromeria fruticosa* oil was characterized by a high content of *γ*-terpinene, *β*-caryophyllene, p-cymene, *α*-pinene, and *β*-bisabolene [67]. These results indicated the possibility of the chemical composition difference in Micromeria EOs from one species to another. The qualitative and/or quantitative difference between the oil composition in our results and those noticed in previous works may be attributed to the ecological factors, genetic differences, environment, geographical origins, and season of harvest [68–72].

As indicated above, *β*-pinene, germacrene D, and *α*-pinene were among the major compounds of TPpEO chemical composition and nerol and *z*-citral were for MGEO. Previous research studies showed the antioxidant effect of *β*-pinene, germacrene D, and *α*-pinene tested individually [73–77]. Also, nerol and citral are known for their antioxidant efficacy [78–80]. These proprieties can explain the antioxidant activity of both essential oils. The small difference of antioxidant activity between TPpEO and MGEO may be associated to the variability in chemical composition since the antioxidant mechanisms of essential oils are generally caused by several compounds' functional groups and their structure [81]. However, the difference observed between testing methods could be explained by the correlation between the chemical composition and/or each compound and the used method [82–84].

For the antibacterial activity, it is known that the Gram-negative bacteria are less sensitive to plant extracts than Gram-positive ones [85–87]. However, the present findings showed that essential oils of plants studied do not have selective antibacterial effects against microorganisms tested. This result may be related to the high level of *β*-pinene, germacrene D, and *α*-pinene (TPpEO) and z-citral and nerol (MGEO). Antibacterial and antifungal activities of these substances have been reported in other studies [75, 88–96]. On the other hand, previous research studies reported the synergic effect of minor compounds against bacteria [97, 98].

## 5. Conclusion

To the best of our knowledge, this is the first report contributing details on chemical composition and antioxidant and antimicrobial activities of *Teucrium polium* subsp. *polium* and *Micromeria graeca* essential oils from Northern Morocco. Our findings have shown that both essential oils are rich by volatile compounds which could be responsible for the observed antibacterial and antioxidant effects. TPpEO and MGEO may be proposed as natural antioxidant and antibacterial product for application on food preservation and management against phytopathogenic bacteria. Further *in vivo* studies will be recommended to investigate their biological proprieties and negative effects before the practical applications.

## Figures and Tables

**Table 1:** Chemical composition of essential oils of *Teucrium polium* subsp*. polium* and *Micromeria graeca* from Northern Morocco.

Compounds	^*∗*^IR	*Teucrium polium* subsp*. polium*	*Micromeria graeca*
		Concentration (% peak area)	
Unknown	—	—	5.43
*α*-Pinene	930	**6.76**	—
*β-*Pinene	975	**19.82**	—
Myrcene	987	2.9	—
1,8-Epoxyl-p-menth-2-ene	988	—	**13.01**
*p-*Cymene	1025	0.55	—
Limonene	1031	**5.71**	—
Unknown	1108	—	2.8
*trans*-Pinocarveol	1145	0.89	—
Camphor	1151	2.45	—
Borneol	1174	1.1	—
Unknown	1184	—	0.47
2-Methyl-1-nonene-3-yne	1194	1.83	—
*z*-Citral	1237	—	**18.25**
Geranial	1266	—	**36.93**
Unknown	1367	—	0.39
*α*-Copaene	1375	0.59	—
Nerol	1376	—	**11.96**
*β*-Bourbonene	1382	1.45	—
Alloaromadendrene	1559	1.02	—
(E),*z*-3-Ethylidenecyclohexane	1465	0.71	—
Germacrene D	1481	**18.33**	-
*β*-Selinene	1488	0.66	—
Bicyclogermacrene	1495	3.21	—
*α*-Muurolene	1497	0.83	—
*α*-Gurjunene	1504	1.13	—
*γ-*Cadinene	1512	1.04	—
*δ*-Cadinene	1517	4.51	—
(*e*)-Farnesene	1560	1.93	—
Spathulenol	1576	4.15	—
Isoaromadendrene epoxide	1581	—	**10.14**
2-Allyphenol	1615	0.68	—
Cadina-1,4-diene	1626	0.56	—
*epi*-Bicyclosesquiphellandrene	1641	**5.05**	—
(-)-Isoledene	1645	1.02	—
Unknown	1652	—	0.57
*α*-Cadinol	1655	**6.83**	—
Italicene	1684	0.76	—
*α*-Elemene	1692	0.99	—
Total:		**97.46**	**90.26**

^*∗*^IR = retention indices relative to C_9_-C_31_ n-alkanes on the DB-5MS capillary column.

**Table 2:** Antioxidant activities of *Teucrium polium* ssp. *polium* and *Micromeria graeca* essential oils.

Assays	Essential oils		Ascorbic acid	Trolox
	*T. polium* subsp*. polium*	*M. graeca*		
DPPH (IC_50_, *µ*g/mL)	208.33 ± 3.51	189.7 ± 2.62	1.82 ± 0.025	1.40 ± 0.04
Reducing power (mg AAE/g of EO)*∗*	1.32 ± 0.10	0.51 ± 0.13	nt	nt

^*∗*^mg AAE/g EO: milligram equivalence of ascorbic acid per gram of essential oil. Values represent mean (standard deviations) for triplicate experiments. nt: not tested.

**Table 3:** Antimicrobial activity of *T. polium* subsp*. polium* and *M. graeca* essential oils using disc diffusion method.

	Inhibition zones diameter (mm)^*∗*^					
	Essential oils			Antimicrobial agents		
	*T. polium* subsp*. polium*	*M. graeca*	Gentamicin (15 *μ*g)	Vancomycin (30 *µ*g)	Streptomycin 25 *µ*g	Amphotericin (10 *μ*g)
*S. aureus CECT 976*	9.66 ± 1.52^+^	22 ± 1^+++^	34.33 ± 0.57	30.66 ± 0.57	nt	nt
*B. subtilis DSM 6633*	23 ± 2^+++^	28.33 ± 1.52^+++^	26 ± 1	27.66 ± 0.57	nt	nt
*L. innocua CECT 4030*	11.33 ± 1.52^+^	19.33 ± 1.15^++^	17.66 ± 0.57	25.33 ± 0.57	nt	nt
*E. coli K12*	10.33 ± 1.52^+^	17.66 ± 1.52^++^	20.33 ± 0.5	8 ± 00	nt	nt
*P. aeruginosa CECT 118*	9.33 ± 0.57^+^	9.33 ± 1.52^+^	19 ± 1	n.e	nt	nt
*P. mirabilis*	21.33 ± 2.08^+++^	20 ± 2^+++^	28.66 ± 0.57	24.33 ± 0.57	nt	nt
*C. michiganensis 1616-3 *	52 ± 1^+++^	>65	nt	nt	24.66 ± 0.57	nt
*P. savastanoi PSS2636-40)*	22 ± 1^+++^	49 ± 1^+++^	nt	nt	26.33 ± 0.57	nt
*C. albicans ATCC 10231*	7.33 ± 0.57^−^	44.33 ± 0.57^+++^	nt	nt	nt	18.66 ± 1.15

^*∗*^The diameter of the inhibition zones (mm), including diameter of disc (6 mm), is given as mean ± SD of triplicate experiments. nt: not tested; n.e: no effect. The sensitivity to the different oils was classified by the diameter of the inhibition halos as follows: not sensitive (−) for diameters less than 8 mm; sensitive (+) for diameters 9–14 mm; very sensitive (++) for diameters 15–19 mm; and extremely sensitive (+++) for diameters larger than 20 mm.

**Table 4:** Minimum inhibitory concentration (MIC) and minimum bactericidal (MBC) or fungicidal (MFC) concentrations mg/ml of essential oils.

Tested microbial strains	Essential oils			
	*Teucrium polium* subsp*. polium*		*Micromeria graeca*	
	MIC	MBC or MFC	MIC	MBC or MFC
*S. aureus CECT 976*	nd	nd	1.56	1.56
*B. subtilis DSM 6633*	0.39	3.12	1.56	>25
*L. innocua CECT 4030*	nd	nd	12.5	12.5
*E. coli K12*	nd	nd	6.25	12.5
*P. aeruginosa CECT 118*	nd	nd	nd	nd
*P. mirabilis*	0.78	1.56	3.12	3.12
*C. michiganensis 1616-3 *	0.78	6.25	0.19	12.5
*P. savastanoi PSS2636-40)*	0.19	0.19	0.78	0.78
*C. albicans ATCC 10231*	nd	nd	3.12	12.5

## Data Availability

The data used in this study are included within the article.

## References

[1] Catherine A. A., Deepika H., Negi P. S. (2012). Antibacterial activity of eugenol and peppermint oil in model food systems. *Journal of Essential Oil Research*.

[2] Kotan R., Çakir A., Ozer H. (2014). Antibacterial effects of *Origanum onites* against phytopathogenic bacteria: possible use of the extracts from protection of disease caused by some phytopathogenic bacteria. *Scientia Horticulturae*.

[3] Isman M. B. (2000). Plant essential oils for pest and disease management. *Crop Protection*.

[4] Balestra G. M., Heydari A., Ceccarelli D., Ovidi E., Quattrucci A. (2009). Antibacterial effect of *Allium sativum* and *Ficuscarica* extracts on tomato bacterial pathogens. *Crop Protection*.

[5] Friedman M., Henika P. R., Mandrell R. E. (2002). Bactericidal activities of plant essential oils and some of their isolated constituents against Campylobacter jejuni, *Escherichia coli*, Listeria monocytogenes, and *Salmonella enterica*. *Journal of Food Protection*.

[6] Burt S. (2004). Essential oils: their antibacterial properties and potential applications in foods-a review. *International Journal of Food Microbiology*.

[7] Chanthaphon S., Chanthachum S., Hongpattarakere T. (2008). Antimicrobial activities of essential oils and crude extracts from tropical *Citrus spp*. against food-related microorganisms. *Songklanakarin Journal of Science and Technology*.

[8] Manniche L. (1999). *Sacred Luxuries: Fragrance, Aromatherapy and Cosmetics in Ancient Egypt*.

[9] Krishna A., Tiwari R., Kumar S. (2000). Aromatherapy-an alternative health care through essential oils. *Journal of Medicinal and Aromatic Plant Sciences*.

[10] Lai P., Roy J. (2004). Antimicrobial and chemopreventive properties of herbs and spices. *Current Medicinal Chemistry*.

[11] Rahman A., Sultana Shanta Z., Rashid M. A. (2016). In vitro antibacterial properties of essential oil and organic extracts of *Premna integrifolia* Linn. *Arabian Journal of Chemistry*.

[12] Hussain A. I., Anwar F., Nigam P. S. (2011). Antibacterial activity of some *Lamiaceae* essential oils using resazurin as an indicator of cell growth. *LWT-Food Science and Technology*.

[13] Ćavar S., Maksimović M., Vidic D., Parić A. (2012). Chemical composition and antioxidant and antimicrobial activity of essential oil of *Artemisia annua* L. from Bosnia. *Industrial Crops and Products*.

[14] Alves-Silva J. M., Dias dos Santos S. M., Pintado M. E., Pérez-Álvarez J. A., Fernández-López J., Viuda-Martos M. (2013). Chemical composition and in vitro antimicrobial, antifungal and antioxidant properties of essential oils obtained from some herbs widely used in Portugal. *Food Control*.

[15] Said Z. B. O. S., Haddadi-Guemghar H., Boulekbache-Makhlouf L. (2016). Essential oils composition, antibacterial and antioxidant activities of hydrodistillated extract of *Eucalyptus globulus* fruits. *Industrial Crops and Products*.

[16] Bajalan I., Rouzbahani R., Pirbalouti A. G., Maggi F. (2017). Antioxidant and antibacterial activities of the essential oils obtained from seven Iranian populations of *Rosmarinus officinalis*. *Industrial Crops and Products*.

[17] Xiang H., Zhang L., Yang Z., Chen F., Zheng X., Liu X. (2017). Chemical compositions, antioxidative, antimicrobial, anti-inflammatory and antitumor activities of *Curcuma aromatica* Salisb. essential oils. *Industrial Crops and Products*.

[18] Siddique S., Parveen Z., e-Bareen F., Mazhar S. (2017). Chemical composition, antibacterial and antioxidant activities of essential oils from leaves of three *Melaleuca* species of Pakistani flora. *Arabian Journal of Chemistry*.

[19] Pattnaik S., Subramanyam V. R., Bapaji M., Kole C. R. (1997). Antibacterial and antifungal activity of aromatic constituents of essentials oils,. *Microbios*.

[20] Wilkinson J. M., Hipwell M., Ryan T., Cavanagh H. M. A. (2003). Bioactivity ofBackhousia citriodora: antibacterial and antifungal activity. *Journal of Agricultural and Food Chemistry*.

[21] Kordali S., Kotan R., Mavi A., Cakir A., Ala A., Yildirim A. (2005). Determination of the chemical composition and antioxidant activity of the essential oil ofArtemisia dracunculusand of the antifungal and antibacterial activities of TurkishArtemisiaabsinthium,A. dracunculus,Artemisia santonicum, andArtemisia spicigeraEssential oils. *Journal of Agricultural and Food Chemistry*.

[22] Güllüce M., Sahin F., Sokmen M. (2007). Antimicrobial and antioxidant properties of the essential oils and methanol extract from Mentha *longifolia L.* ssp*. longifolia*. *Food Chemistry*.

[23] Dambolena J. S., Zunino M. P., López A. G. (2010). Essential oils composition of Ocimum basilicum L. and Ocimum gratissimum L. from Kenya and their inhibitory effects on growth and fumonisin production by Fusarium verticillioides. *Innovative Food Science & Emerging Technologies*.

[24] Nikolić M., Jovanović K. K., Marković T. (2014). Chemical composition, antimicrobial, and cytotoxic properties of five *Lamiaceae* essential oils. *Industrial Crops and Products*.

[25] Piras A., Gonçalves M. J., Alves J. (2018). *Ocimum tenuiflorum L.* and *Ocimum basilicum L*., two spices of Lamiaceae family with bioactive essential oils. *Industrial Crops and Products*.

[26] Bakkali F., Averbeck S., Averbeck D., Idaomar M. (2008). Biological effects of essential oils-a review. *Food and Chemical Toxicology*.

[27] Ben Slimane B., Ezzine O., Dhahri S., Chograni H., Ben Jamaa M. L. (2015). Chemical composition of *Rosmarinus* and *Lavandula* essential oils and their insecticidal effects on *Orgyiatrigotephras(Lepidoptera, Lymantriidae*). *Asian Pacific Journal of Tropical Biomedicine*.

[28] Christofoli M., Costa E. C. C., Bicalho K. U. (2015). Insecticidal effect of nanoencapsulated essential oils from *Zanthoxylum rhoifolium (Rutaceae*) in *Bemisia tabaci* populations. *Industrial Crops and Products*.

[29] Nenaah G. E., Ibrahim S. I. A., Al-Assiuty B. A. (2015). Chemical composition, insecticidal activity and persistence of three *Asteraceae* essential oils and their nanoemulsions against *Callosobruchus maculatus* (F.). *Journal of Stored Products Research*.

[30] Filomeno C. A., Barbosa L. C. A., Teixeira R. R. (2017). *Corymbia* spp. and *Eucalyptus* spp. essential oils have insecticidal activity against *Plutella xylostella*. *Industrial Crops and Products*.

[31] Chellappandian M., Vasantha-Srinivasan P., Senthil-Nathan S. (2018). Botanical essential oils and uses as mosquitocides and repellents against dengue. *Environment International*.

[32] Russo A., Formisano C., Rigano D. (2013). Chemical composition and anticancer activity of essential oils of Mediterranean sage (*Salvia officinalis L*.) grown in different environmental conditions. *Food and Chemical Toxicology*.

[33] Asif M., Yehya A. H. S., Al-Mansoub M. A. (2016). Anticancer attributes of *Illicium verum* essential oils against colon cancer. *South African Journal of Botany*.

[34] Pudziuvelyte L., Stankevicius M., Maruska A. (2017). Chemical composition and anticancer activity of *Elsholtzia ciliata* essential oils and extracts prepared by different methods. *Industrial Crops and Products*.

[35] Bellakhdar J. (1997). *La pharmacopée marocaine traditionnelle. Médecine arabe ancienne et savoirs populaires*.

[36] BenrahmouneIdrissi Z., Dubruille C. (2003). *Invitation à l’amour des plantes. Guide floristique illustré de la réserve biologique de Sidi Boughaba. Scriptura Editions*.

[37] Fougrach H., Badri W., Malki M. (2007). *Flore vasculaire rare et menacée du massif de Tazekka (région de Taza, Maroc)*.

[38] Ennabili A., Gharnit N., El hamdouni E. (2000). Inventory and social interest of medicinal, aromatic and honey-plants from mokrisset region (Nw of Morocco). *Medicinal plants*.

[39] El-Hilaly J., Hmammouchi M., Lyoussi B. (2003). Ethnobotanical studies and economic evaluation of medicinal plants in Taounate province (Northern Morocco). *Journal Ethnopharmacology*.

[40] Khabbach A., Libiad M., Ennabili A., Bousta D. (2012). Medicinal and cosmetic use of plants from the province of Taza, Northern Morocco. *Boletin latinoamericano y del caribe de plantas medicinales y aromaticas*.

[41] Bouyahya A., Abrini J., Et-Touys A., Bakri Y., Dakka N. (2017). Indigenous knowledge of the use of medicinal plants in the North-West of Morocco and their biological activities. *European Journal of Integrative Medicine*.

[42] Costa P., Medronho B., Gonçalves S., Romano A. (2015). Cyclodextrins enhance the antioxidant activity of essential oils from three *Lamiaceae* species. *Industrial Crops and Products*.

[43] Novais M. H., Santos I., Mendes S., Pinto-Gomes C. (2004). Studies on pharmaceutical ethnobotany in arrabida natural park (Portugal). *Journal of Ethnopharmacology*.

[44] Benali T., Khabbach A., Ennabili A., Hammani K. (2017). Ethnopharmacological prospecting of medicinal plants from the Province of Guercif (NE of Morocco). *Moroccan Journal of Biology*.

[45] Ait-Ouazzou A., Lorán S., Bakkali M. (2011). Chemical composition and antimicrobial activity of essential oils of *Thymus algeriensis, Eucalyptus globulus* and *Rosmarinus officinalis* from Morocco. *Journal of the Science of Food and Agriculture*.

[46] Ait-Ouazzou A., Lorán S., Arakrak A. (2012). Evaluation of the chemical composition and antimicrobial activity of *Mentha pulegium, Juniperus phoenicea*, and *Cyperus longus* essential oils from Morocco. *Food Research International*.

[47] El Bouzidi L., Jamali C. A., Bekkouche K. (2013). Chemical composition, antioxidant and antimicrobial activities of essential oils obtained from wild and cultivated Moroccan *Thymus* species. *Industrial Crops and Products*.

[48] Bouyahya A., Et-Touys A., Bakri Y. (2017). Chemical composition of *Mentha pulegium* and *Rosmarinus officinalis* essential oils and their antileishmanial, antibacterial and antioxidant activities. *Microbial Pathogenesis*.

[49] Laghmouchi Y., Belmehdi O., Senhaji N. S., Abrini J. (2018). Chemical composition and antibacterial activity of *Origanum compactum* Benth. essential oils from different areas at northern Morocco. *South African Journal of Botany*.

[50] El-Gharbaoui A., Benítez G., González-Tejero M. R., Molero-Mesa J., Merzouki A. (2017). Comparison of *Lamiaceae* medicinal uses in eastern Morocco and eastern Andalusia and in Ibn al-Baytar’s Compendium of Simple Medicaments (13th century CE). *Journal of Ethnopharmacology*.

[51] Benali T., Bouyahya A., Habbadi K., Zengin G., Khabbach A., Hammani K. (2020). Chemical composition and antibacterial activity of the essential oil and extracts of *Cistus ladaniferus* subsp. *ladanifer* and *Mentha suaveolens* against phytopathogenic bacteria and their ecofriendly management of phytopathogenic bacteria. *Biocatalysis and Agricultural Biotechnology*.

[52] Benali T., Chtibi H., Bouyahya A., Khabbach A., Hammani K. (2020). Detection of antioxidant and antimicrobial activities in phenol components and essential oils of cistus ladaniferus and mentha suaveolens extracts. *Biomedical and Pharmacology Journal*.

[53] Huang B., Ke H., He J., Ban X., Zeng H., Wang Y. (2011). Extracts of *Halenia elliptica* exhibit antioxidant properties *in vitro* and *in vivo*. *Food and Chemical Toxicology*.

[54] Oyaizu M. (1986). Studies on products of browning reaction. Antioxidative activities of products of browning reaction prepared from glucosamine. *The Japanese Journal of Nutrition and Dietetics*.

[55] Rota C., Carramiñana J. J., Burillo J., Herrera A. (2004). *In vitro* antimicrobial activity of essential oils from aromatic plants against selected foodborne pathogens. *Journal of Food Protection*.

[56] Ponce A. G., Fritz R., Del Valle C., Roura S. I. (2003). Antimicrobial activity of essential oils on the native microflora of organic Swiss chard. *LWT-Food Science and Technology*.

[57] Djabou N., Muselli A., Allali H. (2012). Chemical and genetic diversity of two Mediterranean subspecies of Teucrium polium L.. *Phytochemistry*.

[58] El Atki Y., Aouam I., El Kamari F. (2020). Phytochemistry, antioxidant and antibacterial activities of two Moroccan *Teucrium polium* L. subspecies: preventive approach against nosocomial infections. *Arabian Journal of Chemistry*.

[59] Hassan M. M. A., Muhtadi F. J., Al-Badr A. A. (1979). GLC-mass spectrometry of *Teucrium polium* oil. *Journal of Pharmaceutical Sciences*.

[60] Çakir A., Duru M. E., Harmandar M., Ciriminna R., Passannanti S. (1998). Volatile constituents ofTeucrium poliumL. From Turkey. *Journal of Essential Oil Research*.

[61] Kovacevic N. N., Lakusic B. S., Ristic M. S. (2001). Composition of the essential oils of SevenTeucriumSpecies from Serbia and Montenegro. *Journal of Essential Oil Research*.

[62] Aburjai T., Hudaib M., Cavrini V. (2006). Composition of the essential oil from Jordanian germander (Teucrium poliumL.). *Journal of Essential Oil Research*.

[63] Menichini F., Conforti F., Rigano D., Formisano C., Piozzi F., Senatore F. (2009). Phytochemical composition, anti-inflammatory and antitumour activities of four *Teucrium* essential oils from Greece. *Food Chemistry*.

[64] Fertout-Mouri N., Latrèche A., Mehdadi Z., Toumi-Bénali F., Khaled M. B. (2017). Composition chimique et activité antibactérienne de l’huile essentielle de Teucrium polium L. du mont de Tessala (Algérie occidentale). *Phytothérapie*.

[65] Tzakou O., Couladis M. (2001). The essential oil ofMicromeria graeca (L.) Bentham et Reichenb. growing in Greece. *Flavour and Fragrance Journal*.

[66] Duru M. E., Öztürk M., Uğur A., Ceylan Ö. (2004). The constituents of essential oil and in vitro antimicrobial activity of *Micromeria cilicica* from Turkey. *Journal of Ethnopharmacology*.

[67] Formisano C., Mignola E., Rigano D. (2007). Chemical composition and antimicrobial activity of the essential oil from aerial parts ofMicromeria fruticulosa (Bertol.) Grande (Lamiaceae) growing wild in Southern Italy. *Flavour and Fragrance Journal*.

[68] Holm Y., Laakso I., Hiltunen R., Galambosi B. (1998). Variation in the essential oil composition of *Artemisia annuaL*. of different origin cultivated in Finland,. *Flavour and Fragrance Journal*.

[69] Hussain A. I., Anwar F., Nigam P. S., Ashraf M., Gilani A. H. (2010). Seasonal variation in content, chemical composition and antimicrobial and cytotoxic activities of essential oils from four *Mentha* species. *Journal of the Science of Food and Agriculture*.

[70] Duarte A. R., Naves R. R., Santos S. C., Seraphin J. C., Ferri P. H. (2010). Genetic and environmental influence on essential oil composition of *Eugenia dysenterica*. *Journal of the Brazilian Chemical Society*.

[71] Sadeghi H., Jamalpoor S., Shirzadi M. H. (2014). Variability in essential oil of *Teucrium polium* L. of different latitudinal populations. *Industrial Crops and Products*.

[72] Kremer D., Bolarić S., Ballian D. (2015). Morphological, genetic and phytochemical variation of the endemic *Teucrium arduini* L. (*Lamiaceae*). *Phytochemistry*.

[73] Ruberto G., Baratta M. T. (2000). Antioxidant activity of selected essential oil components in two lipid model systems. *Food Chemistry*.

[74] Wang W., Wu N., Zu Y. G., Fu Y. J. (2008). Antioxidative activity of Rosmarinus officinalis L. essential oil compared to its main components. *Food Chemistry*.

[75] Rather M. A., Dar B. A., Dar M. Y. (2012). Chemical composition, antioxidant and antibacterial activities of the leaf essential oil of *Juglans regia* L. and its constituents. *Phytomedicine*.

[76] Aydin E., Türkez H., Geyikoğlu F. (2013). Antioxidative, anticancer and genotoxic properties of *α*-pinene on N2a neuroblastoma cells. *Biologia*.

[77] Shahriari M., Zibaee A., Sahebzadeh N., Shamakhi L. (2018). Effects of *α*-pinene, trans-anethole, and thymol as the essential oil constituents on antioxidant system and acetylcholine esterase of Ephestia kuehniella Zeller (Lepidoptera: Pyralidae). *Pesticide Biochemistry and Physiology*.

[78] Guimarães L. G. D. L., Cardoso M. D. G., Sousa P. E. D., Andrade J. D., Vieira S. S. (2011). Atividades antioxidante e fungitóxica do óleo essencial de capim-limão e do citral. *Revista Ciência Agronômica*.

[79] Sarrou E., Chatzopoulou P., Dimassi-Theriou K., Therios I. (2013). Volatile constituents and antioxidant activity of peel, flowers and leaf oils of *Citrus aurantium* L. Growing in Greece. *Molecules*.

[80] Shi C., Zhao X., Liu Z., Meng R., Chen X., Guo N. (2016). Antimicrobial, antioxidant, and antitumor activity of epsilon-poly-L-lysine and citral, alone or in combination. *Food and Nutrition Research*.

[81] Heim K. E., Tagliaferro A. R., Bobilya D. J. (2002). Flavonoid antioxidants: chemistry, metabolism and structure-activity relationships. *The Journal of Nutritional Biochemistry*.

[82] Tajkarimi M. M., Ibrahim S. A., Cliver D. O. (2010). Antimicrobial herb and spice compounds in food. *Food Control*.

[83] Tounsi M. S., Wannes W. A., Ouerghemmi I. (2011). Juice components and antioxidant capacity of four Tunisian *Citrus* varieties. *Journal of the Science of Food and Agriculture*.

[84] Alam M. N., Bristi N. J., Rafiquzzaman M. (2013). Review on *in vivo* and *in vitro* methods evaluation of antioxidant activity. *Saudi Pharmaceutical Journal*.

[85] Cosentino S., Tuberoso C. I. G., Pisano B. (1999). In-vitro antimicrobial activity and chemical composition of Sardinian Thymus essential oils. *Letters in Applied Microbiology*.

[86] Karaman I., Sahin F., Güllüce M., Ogütçü H., Sengül M., Adigüzel A. (2003). Antimicrobial activity of aqueous and methanol extracts of *Juniperus oxycedrus* L. *Journal of Ethnopharmacology*.

[87] Sahin F., Karaman I., Güllüce M. (2002). Evaluation of antimicrobial activities *Satureja hortensis L*. *Journal of Ethnopharmacology*.

[88] Kim J. M., Marshall M., Cornell J. A., Iii J. F. P., Wei C. I. (1995). Antibacterial activity of carvacrol, citral, and geraniol against *Salmonella typhimurium* in culture medium and on fish cubes. *Journal of Food Science*.

[89] Inouye S., Takizawa T., Yamaguchi H. (2001). Antibacterial activity of essential oils and their major constituents against respiratory tract pathogens by gaseous contact. *Journal of Antimicrobial Chemotherapy*.

[90] Dorman H. J. D., Deans S. G. (2000). Antimicrobial agents from plants: antibacterial activity of plant volatile oils. *Journal of Applied Microbiology*.

[91] Filipowicz N., Kami?ski M., Kurlenda J., Asztemborska M., Ochocka J. R. (2003). Antibacterial and antifungal activity of *juniper berry* oil and its selected components. *Phytotherapy Research*.

[92] Sato K., Krist S., Buchbauer G. (2006). Antimicrobial effect of *trans*-cinnamaldehyde, (−)−Perillaldehyde, (−)−Citronellal, citral, eugenol and carvacrol on airborne microbes using an airwasher. *Biological and Pharmaceutical Bulletin*.

[93] Leite A. M., Lima E. D. O., Souza E. L. D., Diniz M. D. F. F. M., Trajano V. N., Medeiros I. A. D. (2007). Inhibitory effect of beta-pinene, alpha-pinene and eugenol on the growth of potential infectious endocarditis causing Gram-positive bacteria. *Revista Brasileira de Ciências Farmacêuticas*.

[94] Saddiq A. A., Khayyat S. A. (2010). Chemical and antimicrobial studies of monoterpene: Citral. *Pesticide Biochemistry and Physiology*.

[95] Wang W., Li N., Luo M., Zu Y., Efferth T. (2012). Antibacterial activity and anticancer activity of *Rosmarinus officinalis* L. Essential oil compared to that of its main components. *Molecules*.

[96] Wang Y., Zeng X., Zhou Z. (2015). Inhibitory effect of nerol against *Aspergillus niger* on grapes through a membrane lesion mechanism. *Food Control*.

[97] Barel S., Segal R., Yashphe J. (1991). The antimicrobial activity of the essential oil from *Achillea fragrantissima*. *Journal of Ethnopharmacology*.

[98] Tyagi A. K., Malik A. (2011). Antimicrobial potential and chemical composition of *Eucalyptus globulus* oil in liquid and vapour phase against food spoilage microorganisms. *Food Chemistry*.

